# Examination of the Pupils—I

**Published:** 1909-04-10

**Authors:** 


### Medicine.

### EXAMINATION OF THE PUPILS—I.

The best known example of the assistance in diagnosis that may be derived from an examination of the pupils is probably the Argyll-Robertson pupil of locomotor ataxy—a pupil which reacts to accommodation but not to light. It is in consequence of this that a pupillary examination resolves itself, as a rule, into a rapid testing of the light and accommodation reflexes and nothing more. This, however, is a pity, for the behaviour of the pupil is sometimes of considerable diagnostic significance in other diseases; and there should be just as much precision and sequence in the methods of examining the pupils as there is with the heart, lungs, and abdominal organs. It is true that palpation, percussion, and auscultation have no part in any examination of the pupil. Inspection alone must suffice; but the inspection can be made under varying conditions.

In the first place it is important to be sure that any anomaly of the pupils that may be present is not due to any anatomical or pathological lesion in the iris itself. We are at present dealing with the pupil as it is influenced by diseases of the nervous system, and we must therefore assume that steps have been taken to exclude the existence of iritis, glaucoma, keratitis, or other gross ocular lesion. Two allied conditions that deserve particular mention, in order that they may not be accidentally overlooked, are, first, the possibility of atropine having been put into one eye, or both, only a short time previously; and, secondly, the possibility of the patient having a glass eye sufficiently mobile to deceive one if he does not tell one that it is glass.

Assuming that the iris is itself healthy, the first point to note in examining the pupil in a case presenting nervous symptoms is its actual size. It is at the same time important to specify the kind of light the patient's eyes were exposed to at the time. The brighter the light the smaller do the healthy pupils become, and the same sized pupil that might be unnaturally small if it occurred in a dim light might be the natural size for a bright light.

For purposes of reference and comparison it is as well to have the diameter of the pupil stated in absolute figures. It is no very difficult matter to hold a tape measure or other scale close under the eye and read off the size of the pupil in sixteenths of an inch or in millimetres, as the case may be. This will obviate any personal differences there may well be in the use of descriptive adjectives such as "minute," "small," "medium," and "large." Actual measurements of the pupil are easy to make, and the additional accuracy is worth the trouble.

As regards the points that abnormality in the size of the pupil may teach, one may mention first of all the markedly contracted pupils of many cases of locomotor ataxy. It is quite possible, of course, for a person suffering from this disease to have normal-sized or even large pupils; but in most cases of tabes they are small, and sometimes extremely small. A markedly and persistently contracted condition of the pupils, especially in a man of worn, prematurely aged appearance, is highly suggestive of tabes dorsalis, and this quite apart from the use of opium or morphia for the relief of lightning pains.

The other condition which gives rise to markedly contracted pupils is morphinism or the opium habit. The former is readily confirmed by discovery of the needle marks which may be found on various part of the body, especially the left arm in a right-handed person; but the latter is much less easy of detection if the patient chooses to conceal the fact that he takes opium in some form or other habitually. The urine may sometimes serve for the detection of the habit, if it is collected in bulk, concentrated, and tested for the alkaloid.

When the pupils are persistently and markedly dilated a suspicion will arise that the patient is applying some lotion containing atropine or homatropine to them; women sometimes do so with the idea of adding to their personal charms.

In cases of coma, the absolute size of the pupils is sometimes of great diagnostic importance, the two best known conditions that produce both coma and extremely minute or pin-point pupils being acute opium or morphine poisoning and pontine hæmorrhage. The pupils may be fairly small in some other acute conditions, notably in one stage of alcohol poisoning. In opium poisoning a subnormal temperature is to be expected, whereas pontine hæmorrhage very soon causes a rise of temperature or even hyperpyrexia. The further differential diagnosis will depend upon the age and sex of the patient, the history, the condition of the heart and vessels, retinæ, and urine.

Widely dilated pupils in association with delirium or coma are not pathognomonic of any one thing. Many of the causes of coma may give rise to very large pupils, but the largest of all are seen in belladonna poisoning. If accidental, the commonest cause of this is the eating of the cherry-like berries of deadly nightshade by children; in adults acute belladonna poisoning may come about in the same way, but more often it is due to the accidental or suicidal taking of some preparation of atropine or belladonna.

*(To be continued.)*

---

A Paper on the Treatment of Pulmonary Tuberculosis with Ichthyol, modestly, but probably correctly called "hastily-written," was read by Dr. Odell before the recent National Tuberculosis Conference in London. The cases, 123 in number, were treated at the Western Hospital for Incipient Consumption, Torquay, since 1901. No mention is made of controls. The dose given is 7-10 minims thrice daily after food. The results claimed are freedom from hæmoptysis, diminished cough and expectoration, and an increased sense of well-being. It is stated that only one patient died, and this as the result of tuberculous meningitis.

